# Early Motor Developmental Milestones and Personality Traits in Midlife: A 50-Year Follow-Up Study

**DOI:** 10.3390/children10040718

**Published:** 2023-04-13

**Authors:** Trine Flensborg-Madsen, Erik Lykke Mortensen, Jesper Dammeyer, Cathrine Lawaetz Wimmelmann

**Affiliations:** 1 Unit of Medical Psychology, Department of Public Health, University of Copenhagen, 1353 Copenhagen, Denmark; 2 Center for Healthy Aging, University of Copenhagen, 2200 Copenhagen, Denmark; 3 Department of Psychology, University of Copenhagen, 1353 Copenhagen, Denmark

**Keywords:** motor developmental milestones, personality traits, birth cohort, NEO-Five-Factor

## Abstract

***Background*** The purpose of this study was to investigate if infants’ age at attaining motor developmental milestones is associated with the big five personality traits 50 years later. ***Methods*** Mothers of 8395 infants from the Copenhagen Perinatal Cohort recorded a total of 12 motor developmental milestones during the first year of their infant’s life. Information on at least one milestone was available for 1307 singletons with adult follow-up scores on the NEO-Five-Factor Inventory. The mean age at personality testing was 50.1 years. ***Results*** Slower attainment of motor milestones was associated with increased neuroticism and lower conscientiousness in midlife. All 12 motor developmental milestones explained a total of 2.4% of the variance in neuroticism, while they explained 3.2% of the variance in conscientiousness. These results remained significant after adjustment for the included family and perinatal covariates, as well as adult intelligence. ***Discussion*** The personality trait of neuroticism is a general risk factor for psychopathology and has in young adulthood been found to be associated with early motor development. However, evidence on associations of motor developmental milestones with other personality traits has been non-existent. These findings suggest that delays in early motor development may not only characterise individuals with later psychopathology, including schizophrenia, but may also be associated with personality traits such as neuroticism and conscientiousness through the life course.

## 1. Introduction

The child’s development during the first year of life is especially characterised by very prominent and observable motor development [1]. When infants are born, they have very little control over their bodies, but as motor development takes place, the infant is gradually able to, for example, grasp objects with the hands, crawl and walk around without support. Motor development allows the infant to proceed from being fully dependent to being a mobile child with the ability to get around in the environment and interact with objects and other people.

Motor development is a long and complex process, depending on both genetic factors and environmental experiences and opportunities [2]. The ages at which developmental milestones are attained can be understood as indicators of the speed of development, and the maturation of motor skills is related to other domains of neurodevelopment [3,4]. Motor development is part of the psychomotor development referring to changes in a child’s perceptual, cognitive, affective, motor, and social capabilities [2]. Especially during the first year of life, perceptual abilities have been found to be important for motor development [3,4].

The development of motor abilities during the first year of life has theoretically been linked to the infant’s ability to understand the world, with each new motor ability providing new ways for the infant to interact with their surroundings and new ways to gather information and communicate [5,6]. Motor development has thus been found to provide opportunities for the development of perceptual, social and cognitive skills, which makes it likely that it is also related to such skills later in life.

The significance of early-life developmental factors for the development of personality has been proposed in several theories [7,8]. However, the empirical evidence on the importance of specific developmental factors is characterised by a lack of studies following the same individuals from early childhood to adulthood. The hypothesis of developmental precursors of personality has especially been investigated in the Helsinki Birth Cohort Study 1934–1944, where birth weight, length and head circumference were associated with cognitive abilities, temperament, hostility, trait anxiety, depression and attention deficit–hyperactivity disorder symptoms later in life [9,10,11,12]. Additionally, studies from the Copenhagen Perinatal Cohort (CPC) have found that birth weight, head circumference and milestone development in the first three years were associated with cognitive ability in adulthood [13,14].

Infants who are markedly late in achieving developmental milestones during the first years of life have been found to be at higher risk for subsequent diagnoses of some psychiatric disorders. A recent meta-analysis concluded that delayed sitting, standing and walking unsupported were associated with an increased risk of schizophrenia [15]. Additionally, delayed motor development has also been associated with alcohol use disorders [16], as well as psychopathology in general [17].

A high score on the personality trait of neuroticism has been linked to psychopathology in general [18], suggesting that motor development may be associated with this trait. This was investigated in the CPC with adult follow-up ages between 20 and 34 years and using the Eysenck Personality Questionnaire (EPQ), which measures the traits of neuroticism, extraversion and psychoticism. The study concluded that delays in early motor development were associated with neuroticism in young adulthood, while they were not associated with extraversion and psychoticism [19]. Specifically, infants who grew up to have high scores on neuroticism tended to sit without support, crawl, as well as walk later than individuals with low scores on neuroticism in young adulthood. Thus, it can be hypothesised that significant associations between motor development and neuroticism would also be found in midlife.

### The Present Study

The present study was based on the CPC and follows a subsample of this birth cohort until midlife. It thus used a longitudinal design to investigate whether infants’ age at attaining motor developmental milestones is associated with personality traits in midlife. The NEO Five-Factor Inventory (NEO-FFI) was used to measure the big five personality traits: neuroticism, extraversion, openness, agreeableness and conscientiousness.

Based on the previous findings of significant associations between motor development and neuroticism in young adulthood, we hypothesised that the age of attaining milestones in the first year of life would be linked to the personality trait of neuroticism in midlife using the personality measure NEO-FFI. Next, and not previously investigated, it could be hypothesised that motor development is associated with the personality trait of openness to experience, as this personality trait has been linked to intelligence [20].

## 2. Methods

### 2.1. Study Population

The objectives of the study were investigated using the Copenhagen Perinatal Cohort (CPC) and a follow-up study of this cohort: The Copenhagen Aging and Midlife Biobank (CAMB). The CPC was established with data on 8949 mothers and their 9125 infants born at the National University Hospital in Copenhagen between October 1959 and December 1961 [21]. All mothers giving birth in this time period were enrolled, and there were no exclusion criteria. The mean gestational age was 39.2 weeks (SD = 2.0). Information on demographic, socio-economic, prenatal and postnatal factors was recorded prospectively during pregnancy, at delivery and at a 1-year examination. The mothers were mainly residents in Copenhagen, but some were also admitted due to obstetrical complications or single-mother status [22]. At the time of investigation, the following medical indications gave access to delivery at Rigshospitalet: complications in pregnancy, anticipated complicated delivery, previously complicated pregnancies, the mother being older than 35 years of age, and social indications including single mothers or mothers in poor social conditions. The cohort was thus selected and characterised by a higher frequency of complications and a higher incidence of single mothers than in the general population [23]. However, the vast majority of the mothers and children were characterised as representative of the Danish population at the time of investigation, which has been previously described [23]. A total of 8395 infants were alive the first month after birth.

During the time span from 2009 to 2011, 5282 individuals from the CPC were invited to participate in the CAMB 50-year assessment. In this subsample, data on the NEO Five-Factor Inventory (NEO-FFI) were available for 1705 (32.3%) individuals, and among these participants, information on at least one motor developmental milestone was available for 1346 individuals. A total of 39 twins were excluded, whereby the final sample included 1307 singletons, of whom, 582 were men and 725 were women. The mean age was 50.1 years (range: 48.5–51.4) at the time when personality was tested.

### 2.2. Motor Developmental Milestones

Developmental milestones were obtained from the mothers who were instructed to use a standardised diary to record the ages at which their child reached each of 12 developmental milestones. At a 1-year examination, the diary was brought to the hospital [21], and if the mother had not completed the diary, an effort was made to obtain retrospective information. Table 1 shows the recorded milestones during the first year of life, including the median and quartile ages at attainment for each. The 1% and 99% percentiles of each milestone were used as cut-offs for valid milestones, and more extreme milestone values were rescored to the smallest and highest values in the range defined by the 1% and 99% percentiles. For comparison, windows of normal variation in milestones have been described by the WHO [24].

In the study sample, the rate of missing data on individual milestones among participants varied from 11.2% (standing with support) to 61.4% (walking without support). To reduce the influence of missing data and based on the assumption that the means of milestone ratings would be more reliable than individual milestone ratings, composite milestone means were derived previously by a principal component analysis [14]. The expectation–maximisation (EM) algorithm [25] was used to conduct the principal component analysis, in which a dataset with missing milestone data replaced by imputed data was constructed. The analysis of this imputed dataset showed that the first three components explained 67% of the variance, and both varimax and promax rotation defined three factors: (a) smiling and lifting head (milestones 1–3); (b) rolling, crawling, sitting and grabbing (milestones 4–8); and (c) standing and walking (milestones 9–12). To derive composite factor milestone means for the three factors and the overall mean of the 12 1-year milestones, we linearly standardised the age of attainment of each milestone to a mean of 0 and a standard deviation of 1. The mean scores were then calculated as the mean of the included individual milestone scores. The imputed dataset was not used to calculate means; thus, the mean of the available milestone scores was calculated if data were missing on one or more milestones included in a mean. Finally, the four means were re-standardised to a mean of 0 and a standard deviation of 1.

### 2.3. NEO-Five-Factor Inventory

The NEO-FFI is a Danish shortened version of the Revised NEO personality inventory (NEO-PI-R) [26]. It assesses the big five traits: neuroticism, extraversion, openness, agreeableness and conscientiousness, and it is based on 12 items for each trait. These 60 items are scored on a scale ranging from 0 = strongly disagree to 4 = strongly agree, resulting in a total score range for each trait of 0–48 and with Cronbach’s alpha ranging between 0.69 (agreeableness) to 0.85 (neuroticism) [27]. Factor score correlations between NEO-PI-R and NEO-FFI factor scores are in the range of 0.89 to 0.93, and the psychometric properties of NEO-FFI have been considered to be good [28,29,30,31].

### 2.4. Covariates

The following covariates were considered potential confounding factors: sex of the child, parental socio-economic status (SES), parity, mother’s age, father’s age, single-mother status and birth weight. The selection of covariates was based on theoretical considerations of each variable to be associated with both motor developmental milestones and personality while not being an intermediate variable on the possible causal pathway.

Information on parity and single-mother status was obtained from interviews during pregnancy by a physician, A.L. Villumsen (1970), who interviewed all the women. Information on the sex of the child and birth weight was obtained from the postnatal examination of the mother and the child, while information on parental SES (on a 1–8 point scale) was obtained from a 1-year examination of the mother and child. Information on maternal and paternal age was obtained from the Danish Civil Registration System.

We included intelligence as a covariate in supplementary analyses as it has been found to be associated with both motor developmental milestones [13] and personality [23]. Intelligence was measured by the Intelligenz-Struktur-Test (I-S-T 2000 R) [32] (translated into Danish by Hogrefe Publishers) using three subtests (sentence completion, verbal analogies and number series). It was administered as part of the CAMB 50-year data collection.

All variables were included as linear continuous variables except for sex and parity, the latter of which was included as a binary variable indicating first or later pregnancy).

### 2.5. Statistical Analyses

In descriptive Table 2, a median split was applied to continuous covariates, and independent samples *t*-tests were used to test the mean differences between the two subgroups in both the overall mean of milestones and the mean level of neuroticism (Table 2). Associations of each motor developmental milestone and the milestone means with each personality trait were estimated in linear regression analyses in both unadjusted and fully adjusted models (in these models, continuous covariates were analysed as linear variables). The purpose of the analyses was to obtain unbiased estimates and not to estimate effects associated with each covariate, wherefore collinearity among the covariates was not considered a substantial problem. Analyses were conducted using full information maximum likelihood (FIML) analyses [33], in which we used the structural equation modelling facilities of Stata 14 (StataCorp LP, College Station, TX, USA) to utilise all available information, including that of participants with missing data on milestones or covariates. Preliminary analyses tested the interaction of the overall mean of milestones with sex and parental SES for all personality traits. No significant interactions were found. Additional analyses including intelligence as a covariate were conducted. According to Danish laws, approval to conduct the present study does not require permission by the scientific ethical committee system.

## 3. Results

The last attained milestones were ‘standing without support’ and ‘walking without support’, which had a median age at attainment of 10.5 and 11.5 months, respectively (Table 1).

The results in Table 2 show that parity, mother’s age, father’s age, single-mother status and birth weight were all significantly associated with the overall mean of the 12 milestones. Only sex was significantly associated with neuroticism in adulthood.

Most of the significant associations between motor developmental milestones and personality traits were found for neuroticism and conscientiousness (Table 3). For neuroticism, a significant positive association was found for ‘walking without support’ (adjusted β = 0.09, *p* < 0.05). Thus, the slower attainment of this milestone was associated with increased neuroticism in midlife. Additionally, a positive association was found for the mean of all milestones (adjusted β = 0.07, *p* < 0.05), implying that a general delay in milestone attainment is associated with increased neuroticism later in life.

For conscientiousness, a significant negative association was found for ‘standing with support’ (adjusted β = −0.08, *p* < 0.01), implying that the slower attainment of this milestone was associated with decreased conscientiousness in midlife. Additionally, a negative association was found for the milestone mean of standing and walking (adjusted β = −0.07, *p* < 0.05) and the overall mean of milestones (adjusted β = −0.06, *p* < 0.05). When included in the same model, the 12 motor developmental milestones explained 2.4% and 3.2% of the variance in neuroticism and conscientiousness, respectively.

For the personality traits of extraversion, openness and agreeableness, few significant associations were found, and significantly adjusted associations were only found for agreeableness. These were found for the milestone ‘sitting without support’ and the mean rolling, crawling, sitting and grabbing, with positive associations indicating that the slower attainment of these milestones was associated with lower agreeableness scores in midlife.

Supplementary analyses including intelligence as a covariate generally showed the same patterns as those shown in Table 3; thus, associations of milestones with personality traits were largely independent of intelligence scores.

## 4. Discussion

### 4.1. Main Results

The study confirmed our hypothesis that the slower attainment of motor developmental milestones in the first year of life was associated with higher neuroticism in midlife. Thus, significant associations were found for the milestone ‘walking without support’ in addition to the overall mean of milestones. Motor developmental milestones explained 2.4% of the variance in neuroticism in midlife. Our hypothesis of motor development being associated with openness was not confirmed, as none of the adjusted estimates were significant. However, there were significant associations between motor development and conscientiousness, and 3.2% of the variance in conscientiousness in midlife was explained by motor developmental milestones. Additionally, the results indicated associations between the faster attainment of milestones and higher agreeableness.

### 4.2. Comparison with Other Studies

As far as we are aware, this is the first study to investigate associations between motor development in the first year of life and the big five personality traits, and it is the first study to explore associations of motor development with personality traits in midlife. Nevertheless, a previous study based on the same birth cohort found significant associations between the faster attainment of motor developmental milestones and a lower level of neuroticism, measured with the EPQ, in young adulthood. Additionally, it found a beta value of 0.10 (*p* < 0.01) for the mean of all milestones [23], which is very comparable to the results in the present study, in which the beta value was 0.07 (*p* < 0.01) for the mean of all milestones in relation to neuroticism. Additionally, in the previous study, a total of 2.8% of the variance in neuroticism scores in young adulthood was explained by the 12 included milestones [23], which is comparable to 2.4% of the variance explained in the present study. This suggests both that the association between motor development and neuroticism is significant across the life course and that associations exist with both the EPQ and NEO–FFI.

Significant associations with neuroticism are additionally supported by former studies on motor development and psychopathology that found later ages of standing and walking to be related to the risk of schizophrenia. A review thus concluded that the following milestones were significantly associated with the adult risk of schizophrenia: sitting unsupported, standing unsupported and walking unsupported [15]. These milestones correspond to those that had the highest estimates with neuroticism in the present study. For example, walking without support was significantly associated with neuroticism with a beta coefficient of 0.9 (*p* < 0.05). Other studies suggest that the late attainment of motor milestones is not necessarily specific to the psychopathology of schizophrenia but is also associated with alcohol use disorders [16] and with other psychiatric disorders in general [17]. Additionally, neuroticism has been suggested to be a risk factor of schizophrenia [34,35] in addition to being linked with a generally increased risk of psychopathology [18,36,37]. Therefore, our findings are in agreement with studies on motor development and psychopathology and suggest that delayed milestones may be an early key phenotype associated with both adult personality traits and psychopathology. Alternatively, neuroticism may mediate the link between milestones and psychopathology.

Associations between motor development and personality traits other than neuroticism have not previously been found. However, significant associations have been found between faster motor development and intelligence in young adulthood [13,14] and midlife [23]. We therefore hypothesised we would find associations between motor development and openness, as high correlations are often found between this personality trait and intelligence [20], but this hypothesis was not supported by the results.

### 4.3. Interpretation

A number of mechanisms are likely to explain why associations exist between motor development in the first year of life and personality 50 years later. Firstly, there may be a causal effect of motor development on personality, in which the timing of specific motor developmental abilities affects the development of certain personality traits. For example, children who learn to walk earlier have increased opportunities to engage in their surroundings, which may decrease their tendency to develop facets related to anxiety or depression or increase the opportunity to develop facets related to conscientiousness such as competence and achievement striving. A causal explanation linking motor development to neuroticism is supported by a study that found lower motor performance at 3 and 30 days to be associated with negative affectivity at 4 months [38]. Secondly, associations may reflect reverse causality, whereby early characteristics related to personality may affect the timing of motor development. This explanation does not, however, seem plausible, as specific personality traits are not distinctive in early childhood [39]. Thirdly, common causes may affect both motor milestone development and personality traits. Such potential factors include genetic factors in addition to proximal factors in the home environment, such as parent−child interaction [40,41,42,43,44,45]. For example, some motor delays could be related to generic neurological or sensory impairment, and these aspects could potentially also affect temperament. Furthermore, temperament in early childhood is associated with later personality traits [46,47], whereby specific aspects of temperament combine to define the constructs of extraversion, negative affect and effortful control [48,49]. Thus, there might be a dynamic interplay between early motor development and temperament that explains the associations with personality traits found in this study.

Only the later milestones such as sitting without support, standing and walking were significantly associated with personality in adjusted analyses. These all require complex motor coordination and may to a higher degree develop as a result of interaction between the child and the parents. It is thus plausible that parent–child dynamics are more central to the attainment of later than earlier milestones, which may explain the associations with later motor milestones and personality traits. Thus, children with a secure attachment between 12 and 18 months have been found to score lower on neuroticism and higher on agreeableness and conscientiousness in adulthood [50].

### 4.4. Methodological Issues

The main advantage of this study is the prospective design, including the real-time documentation of milestone attainment by the mothers in the children’s first year of life and a 50-year follow-up with well-validated measures of personality.

The frequency of missing data tended to be high, especially for standing and walking without support. This may reflect that these milestones may not have been attained by some children at the time of the 1-year follow-up and therefore were not recorded. As this source of missing data primarily concerns late-developing children, systematic selection bias may have attenuated the observed associations between these later milestones and personality traits, with the present results most likely underestimating the associations.

As described, the CPC is based on a birth cohort that comprises all births at a general university hospital in Denmark. However, it is still to some extent a selected cohort, and moreover, the follow-ups are characterised by a higher proportion of individuals with high parental SES. There are, however, no obvious reasons as to why associations between early predictors and personality should be different in non-participants, and thereby, it is not very plausible that selection bias would have occurred. However, only a total of 32% of invited members of the CPC participated in the CAMB follow-up. The follow-up sample differed from the full cohort in terms of higher infant SES (the mean SES at the 1-year follow-up examination was 4.0 for the full cohort, while it was 4.6 in the present study subsample). The variance in certain personality traits may therefore have been reduced (e.g., lower neuroticism scores). However, no interactions were found between the mean of milestones and infant SES; therefore, selection bias was not considered to be a substantial problem in the present study.

A potential limitation is the possibility of type 1 errors. Thus, statistical tests were conducted on 12 milestones for five personality traits. However, due to the fact that our findings are in agreement with the initial hypotheses and previous findings, especially those for neuroticism, type 1 errors may not be a substantial problem. This is additionally supported by the fact that only the later milestones were associated with personality traits, as this indicates a consistent pattern and thus a non-incidental finding.

As this is an observational study, there may be unrecognised confounding variables; these may be related to genetic factors affecting brain development or nutrient levels during pregnancy and in the first years of life. In addition, proximal factors in the home environment, including the mental resources of the parents, may also have affected both motor development and the development of personality.

Finally, due to the follow-up time of 50 years, the results are based on individuals that were born in the first half of the second part of the last century. Obviously, children’s environments have changed substantially, but it is unclear how such changes may influence associations between motor development and later personality.

## 5. Conclusions

The results of this study showed that motor development during the first year of life has small but significant associations with several personality traits in midlife. More specifically, the faster attainment of motor developmental milestones was associated with lower neuroticism and higher conscientiousness and agreeableness. The variance in these personality traits explained by motor development varied between 1.2 and 3.2%.

This study contributes to the existing literature on possible influences of the timing of motor development, and it suggests a link between motor development and personality 50 years later. Whether these associations reflect a direct effect of motor development on the development of personality or confounding factors cannot be determined from this study. The mechanisms explaining these results may additionally be different for each personality trait.

As this is the first study to investigate motor developmental milestones in relation to personality traits in midlife, more research should be conducted before discussions of clinical implications will be appropriate. Future research should thus address the mechanisms, including potential confounding factors. However, the associations in this study demonstrate lifelong associations between motor development and individual differences in personality.

## Figures and Tables

**Table 1:** Descriptive characteristics of motor developmental milestones.

	Developmental Milestones		Description	N	Median (Months)	25/75% Percentiles
Smiling and lifting head	1	Lifts head on stomach (weeks)	The child can lift the head when placed on the stomach (weeks)	1049	3	2/5
(N = 1172)	2	Smiles (weeks)	The child can smile (weeks)	1098	5	4/7
	3	Holds head when sitting	The child holds the head when pulling arms to a sitting position	989	3	2.5/4
Rolling, crawling, sitting and grabbing (N = 1263)	4	Grasps after things	The child grasps after things and holds on to them	983	4	3/4.5
	5	Rolls	The child rolls from back to stomach	979	6	5/7
	6	Sits without support	The child can sit without support	1189	7	6/8
	7	Crawls	The child can crawl	879	9	8/10
	8	Crawls longer distance	The child can crawl a longer distance (e.g., across the living room)	776	9.25	8/10.5
Standing and walking (*n* = 1249)	9	Stands with support	The child can stand when supported	1160	8.5	7.5/10
	10	Stands without support	The child can stand unsupported	644	10.5	9/11
	11	Walks with support	The child can walk when supported	1013	10	9/11
	12	Walks without support	The child can walk unsupported	504	11.5	11/12

**Table 2:** Associations of covariates with the overall mean of milestones and neuroticism.

Covariates ^b^	N	Mean of Milestones	*p* ^a^	Mean Level of Neuroticism	*p* ^a^
Sex of the child					
Boy	579	0.001	0.98	16.4	<0.001
Girl	725	−0.001		19.4	
Parental SES					
Lower end (<4)	583	−0.05	0.34	18.3	0.34
Higher end (≥4)	544	0.002		17.9	
Parity (first child)					
Yes	615	−0.16	<0.001	18.0	0.92
No	692	0.14		18.1	
Mother’s age					
<24.5 years	674	−0.09	0.001	17.8	0.27
≥24.5 years	628	0.10		18.3	
Father’s age					
<28.5 years	642	−0.13	<0.001	17.8	0.41
≥28.5 years	638	0.12		18.2	
Single mother					
Yes	279	−0.17	0.001	17.9	0.72
No	1024	0.04		18.1	
Birth weight					
<3300 g	653	0.14	<0.001	18.3	0.18
≥3300 g	648	−0.15		17.8	
Intelligence					
<103	664	0.03	0.33	18.4	0.11
≥103	631	−0.03		17.7	

^a^*t* test. ^b^ Covariates in this table were divided to achieve approximately 50% in each category.

**Table 3:** Standardised regression coefficients for milestones predicting level of personality traits (SEM).

	Neuroticism		Extraversion		Openness		Agreeableness		Conscientiousness	
Developmental Milestones	β	β^adjusted^	β	β^adjusted^	β	β^adjusted^	β	β^adjusted^	β	β^adjusted^
Lifts head on stomach	0.06	0.05	0.01	0.01	−0.01	−0.001	0.02	0.01	−0.01	−0.003
Smiles	0.03	0.02	0.004	0.01	−0.01	−0.02	0.02	0.01	−0.01	−0.01
Holds head when sitting	0.003	0.01	0.03	0.04	0.002	0.02	0.04	0.06	−0.01	−0.01
Grasps after things	0.02	0.02	−0.06 *	−0.06	−0.05	−0.05	0.04	0.05	−0.02	−0.03
Rolls	−0.001	−0.002	−0.01	−0.002	−0.01	−0.02	−0.01	−0.002	0.02	0.01
Sits without support	0.02	0.04	−0.01	−0.02	−0.001	−0.03	0.03	0.07 *	0.004	−0.01
Crawls	0.03	0.01	0.04	0.04	0.05	0.04	0.05	0.03	0.001	−0.01
Crawls longer distance	0.04	0.01	0.03	0.04	0.04	0.02	0.03	0.02	0.02	0.02
Stands with support	0.06	0.04	−0.05	−0.05	−0.01	−0.003	0.04	0.02	−0.08 **	−0.08 **
Stands without support	0.07	0.07	−0.04	−0.04	0.09 *	0.08	0.005	−0.003	−0.06	−0.07
Walks with support	0.04	0.03	−0.04	−0.04	0.05	0.05	0.04	0.04	−0.03	−0.03
Walks without support	0.09 *	0.09 *	0.02	0.03	0.07	0.06	0.03	0.02	−0.09 *	−0.09
**Milestone means:**										
Smiling and lifting head	0.04	0.04	0.02	0.04	−0.01	0.001	0.02	0.01	−0.02	−0.02
Rolling, crawling, sitting and grabbing	0.03	0.03	−0.01	−0.01	−0.01	−0.02	0.04	0.06 *	−0.01	−0.02
Standing and walking	0.06 *	0.05	−0.02	−0.02	0.03	0.03	0.04	0.03	−0.07 *	−0.07 *
Overall mean of milestones	0.07 **	0.07*	−0.02	−0.01	0.02	0.01	0.05	0.05	−0.05	−0.06 *
Explained variance of 12 milestones:	2.4%		2.0%		1.9%		1.2%		3.2%	

*: <0.05; **: <0.01. Adjusted for: sex, parental socio-economic status, parity, mother’s age, father’s age, single-mother status, and birth weight.

## Data Availability

Data may be available upon request from the relevant steering committees.
